# Exploring the endocrine activity of air pollutants associated with unconventional oil and gas extraction

**DOI:** 10.1186/s12940-018-0368-z

**Published:** 2018-03-21

**Authors:** Ashley L. Bolden, Kim Schultz, Katherine E. Pelch, Carol F. Kwiatkowski

**Affiliations:** 1 The Endocrine Disruption Exchange (TEDX), www.TEDX.org, Eckert, Colorado USA; 2 Department of Integrative Physiology, University of Colorado, Boulder, Colorado USA; 3 Biological Sciences, North Carolina State University, Raleigh, North Carolina USA

**Keywords:** Endocrine disruption, Unconventional oil and gas, Hydraulic fracturing, Fracking, Air pollutants, Reproduction, Neurological, Developmental, Hormone

## Abstract

**Background:**

In the last decade unconventional oil and gas (UOG) extraction has rapidly proliferated throughout the United States (US) and the world. This occurred largely because of the development of directional drilling and hydraulic fracturing which allows access to fossil fuels from geologic formations that were previously not cost effective to pursue. This process is known to use greater than 1,000 chemicals such as solvents, surfactants, detergents, and biocides. In addition, a complex mixture of chemicals, including heavy metals, naturally-occurring radioactive chemicals, and organic compounds are released from the formations and can enter air and water. Compounds associated with UOG activity have been linked to adverse reproductive and developmental outcomes in humans and laboratory animal models, which is possibly due to the presence of endocrine active chemicals.

**Methods:**

Using systematic methods, electronic searches of PubMed and Web of Science were conducted to identify studies that measured chemicals in air near sites of UOG activity. Records were screened by title and abstract, relevant articles then underwent full text review, and data were extracted from the studies. A list of chemicals detected near UOG sites was generated. Then, the potential endocrine activity of the most frequently detected chemicals was explored via searches of literature from PubMed.

**Results:**

Evaluation of 48 studies that sampled air near sites of UOG activity identified 106 chemicals detected in two or more studies. Ethane, benzene and n-pentane were the top three most frequently detected. Twenty-one chemicals have been shown to have endocrine activity including estrogenic and androgenic activity and the ability to alter steroidogenesis. Literature also suggested that some of the air pollutants may affect reproduction, development, and neurophysiological function, all endpoints which can be modulated by hormones. These chemicals included aromatics (i.e., benzene, toluene, ethylbenzene, and xylene), several polycyclic aromatic hydrocarbons, and mercury.

**Conclusion:**

These results provide a basis for prioritizing future primary studies regarding the endocrine disrupting properties of UOG air pollutants, including exposure research in wildlife and humans. Further, we recommend systematic reviews of the health impacts of exposure to specific chemicals, and comprehensive environmental sampling of a broader array of chemicals.

**Electronic supplementary material:**

The online version of this article (10.1186/s12940-018-0368-z) contains supplementary material, which is available to authorized users.

## Background

Advanced techniques used to develop oil and gas resources, including horizontal drilling and hydraulic fracturing (fracking), have unlocked fossil fuels from formations previously unavailable for extraction, including shale and tight sands. Research has found that unconventional oil and gas (UOG) development and production is associated with air pollution [1–7], contamination of surface, ground, and drinking water [8–10], as well as soil and sediment contamination [11–13]. Contaminants released from UOG sites enter the air readily during well pad development and continue for the life of the well, impacting both local and regional air quality. Industry wide there are hundreds of different products composed of a mixture of chemicals used during drilling, fracturing, and the cleaning and maintenance of well pads and equipment. Many of them are volatile and include several known carcinogens and hazardous air pollutants (HAPs) listed under the Clean Air Act [14]. Air pollutants are released both from the products and mobile and stationary equipment commonly used during UOG operations [6, 15, 16]. Further, unprocessed natural gas contains many volatile compounds that surface with methane and are released to the environment through venting and flaring and through fugitive emissions from well pipe fittings and equipment [6, 16–18]. Additionally, open evaporation pits that contain fracking fluids that return to the surface (flowback) and water produced from fracturing the formation (produced water) further impact air quality in these areas [19–21]. Due to the potential for wide-spread exposure to air pollutants released from UOG activity and the growing number of oil and gas wells being drilled in close proximity to neighborhoods, including schools and recreational areas, the health of nearby communities may be at risk. Indeed, several studies have shown that UOG activity may adversely impact the health of humans and animals [22–26] and the environment [27–29].

These concerns have led to a growth in epidemiologic research with many studies suggesting a link between UOG proximity and adverse health impacts. Self-reported symptoms by Pennsylvania residents living near UOG operations in the Marcellus Shale include impacts to the upper respiratory system, irritation of the skin and sensory organs, and increased headaches [25, 30]. Additional studies also considered well activity or density, a method used to estimate exposure to air pollutants. McKenzie et al., found an increased risk of neurological and respiratory effects, blood disorders, and adverse developmental outcomes in Colorado residents living within one-half mile of natural gas wells [31]. These observations were more pronounced during well completion activities [31]. Increased odds of asthma exacerbations [32], nasal irritation, migraine headaches, and fatigue symptoms were more often reported by residents living near sites with higher UOG activity compared to a control population [33]. Risk of childhood hematologic cancer was also increased with increased density of UOG wells [34]. Further, retrospective cohort studies have linked UOG activity to adverse reproductive and developmental outcomes, such as preterm birth [35, 36], low birth weight [37], congenital anomalies [38], and infant mortality [36, 39]. These outcomes suggest a possible relationship between maternal exposure to endocrine disrupting chemicals and birth outcomes; however, results across studies are mixed.

In addition to epidemiological studies, recent studies using *in vitro* and experimental animal models to assess the connection between UOG activity and endocrine-related outcomes have been published. In these initial studies chemicals detected in water collected near UOG operations such as spill sites and surface water near wastewater injection sites were shown to have activity in estrogen, androgen, progesterone, glucocorticoid, and thyroid hormone *in vitro* receptor assays [10, 40]. In laboratory experiments exposure has resulted in similar impacts across several different models. Specifically, male rodents exposed prenatally to a mixture of chemicals used during hydraulic fracturing were shown to have increased organ weights of the testes and thymus, decreased sperm counts, and increased serum testosterone levels [41]. Effects in female rodents included hormone suppression, changes in uterine, ovary, heart, and body weights, and disrupted folliculogenesis [42]. Emerging research in zebrafish embryos found that exposure to flowback/produced water from UOG increased embryo deformations and mortality, reduced metabolic rates, and altered cardio-respiratory gene expression [43, 44]. Further, embryonically exposed juveniles demonstrated decreased metabolic rates and fitness as judged by swim performance [45]. In exposed juvenile rainbow trout mRNA expression was elevated for several genes including vitellogenin and estrogen receptor alpha 2. Additionally, expression of oxidative stress and biotranformation genes in the liver and gills was observed [46]. Finally, exposure of *Daphnia* to flowback/produced water resulted in decreased reproduction and altered gene expression [47].

The purpose of this evaluation was to employ systematic screening-level methods to begin to prioritize air pollutants associated with UOG that have evidence of endocrine activity. This work could be used to identify avenues for primary research to understand endocrine disrupting properties of air pollutants; provide the groundwork for in-depth reviews of the health impacts of exposure to specific chemicals (i.e., systematic or scoping reviews); offer rationale for further exposure research in wildlife and humans; and lastly, identify research gaps. Specifically, two objectives were completed; 1) identification of the most commonly detected chemicals in the air near UOG activity, as reported in original research, and 2) to determine if this subset of air pollutants has been shown to have endocrine activity or have effects that could be linked to disrupted endocrine signaling.

## Methods

### Identification of air pollutants near sites of UOG activity

Comprehensive literature searches were performed in order to identify studies that measured compounds in air near or on sites of UOG development in the United States (US). We used Web of Science and PubMed to complete electronic searches for all years to June 2016. The search logic was developed using terms for major geologic formations in the US where UOG activity occurs and terms that linked the formations to air emissions (Additional file 1: Table S1). The titles and abstracts of these articles were then screened for relevance using Distiller SR® [48] by two independent reviewers. For inclusion, studies had to present primary findings, be in the English language, and measure air pollutants near sites of UOG production. Studies that only measured methane were excluded. Discrepancies regarding inclusion were discussed and resolved by the two reviewers. Summary level data from relevant studies were collected. Parameters included publication date, chemicals detected, and the location of measurement. These data were used to develop the list of compounds detected in air. This initial list was then used to yield a list of the chemicals detected in greater than 10 UOG air sampling studies.

### Determination of endocrine activity of UOG related air pollutants

The list of air pollutants associated with UOG production ascertained from peer-reviewed literature was cross-referenced with the Endocrine Disruption Exchange (TEDX) List of Potential Endocrine Disruptors (http://endocrinedisruption.org/interactive-tools/tedx-list-of-potential-endocrine-disruptors/search-the-tedx-list: accessed October 2016) to determine if any of the chemicals had been characterized as having endocrine activity [49]. The TEDX List of Potential Endocrine Disruptors is a database that contains expert verified citations illustrating evidence of endocrine disruptive properties of a variety of chemicals; this database is continually updated as new evidence about chemicals becomes available [49]. Cross-referencing yielded the initial list of chemicals with evidence of endocrine activity. For this initial list, citations from the TEDX List of Potential Endocrine Disruptors were used as evidence of endocrine activity. We then performed searches in PubMed using the chemical name and CAS number for the remaining chemicals detected in greater than 10 UOG air sampling studies to determine whether or not those chemicals had evidence documented in the peer-reviewed literature regarding their potential endocrine activity (for the individual chemical search terms see Additional file 1: Table S2). The following 15 chemicals were searched in PubMed: ethane, n-pentane, propane, n-butane, isopentane, isobutane, m,p-xylene, o-xylene, ethylene, methylcyclohexane, n-heptane, acetylene, n-octane, propylene, and cyclohexane. The PubMed records were imported into Sciome Workbench for Interactive computer-Facilitated Text-mining (SWIFT)-Review [50] and filtered using search terms (modified from [51, 52]) intended to identify articles that assessed the endocrine activity of the compounds (see Additional file 1: Table S3). Though xylenes (the isomeric mixture) is listed on the TEDX List of Potential Endocrine Disruptors we performed searches for the compounds as represented in the air sampling studies (i.e., m,p-xylene and o-xylene). In addition, studies that evaluated the effects of exposure to m-xylene and p-xylene separately and citations from the TEDX List of Potential Endocrine Disruptors that assessed the xylenes were included.

## Results

Our search of the literature from PubMed and Web of Science yielded 1366 and 2907 potential records, respectively (including any duplicate records). Screening of titles and abstracts by two reviewers identified 97 relevant articles. Full text review of the articles yielded 43 inclusions and 54 exclusions (30 duplicates, five that did not assess specific chemicals, 10 reviews, four conference abstracts, and five categorized as other [e.g., methods development]). In addition, hand searching yielded five other studies that met inclusion criteria, resulting in a total of 48 included studies.

**Fig. 1:**
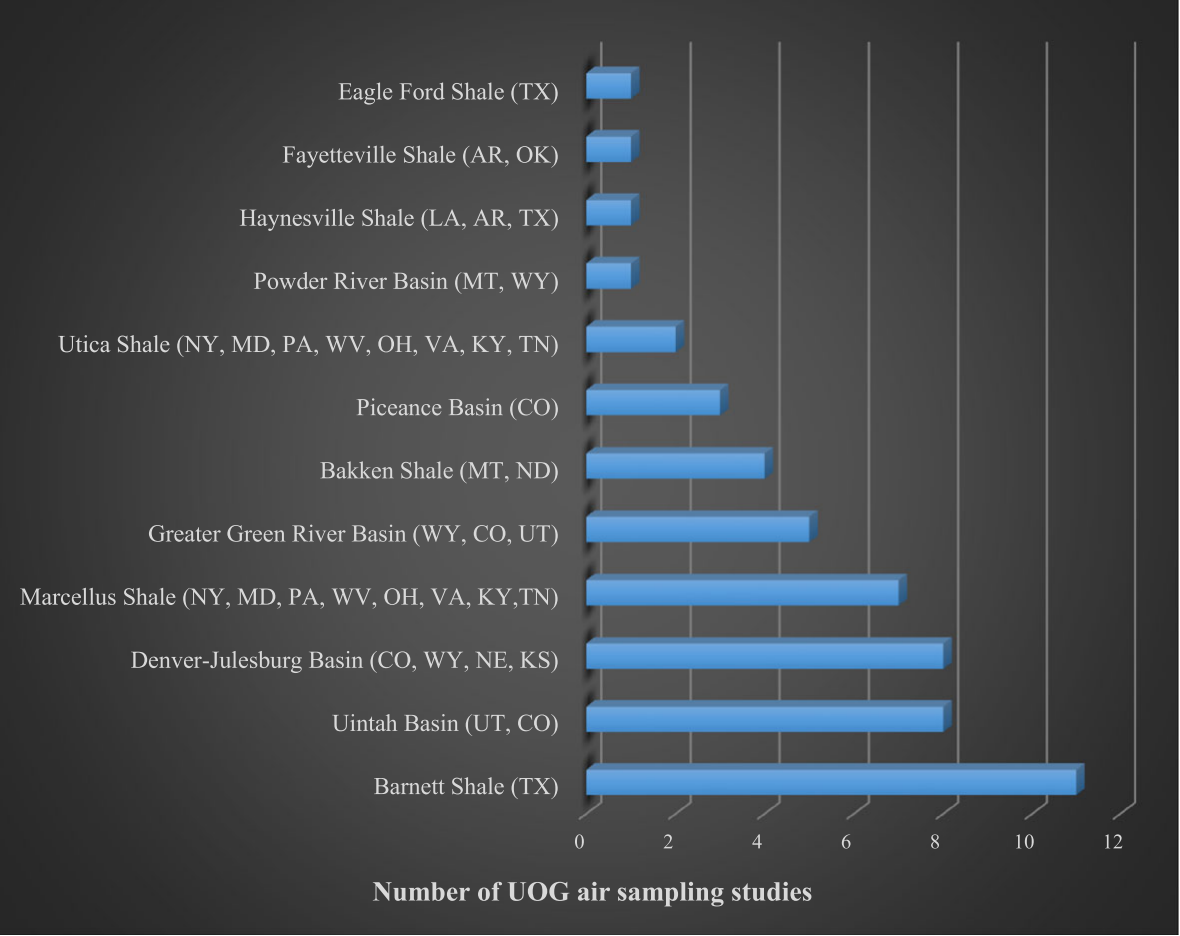
Number of UOG air sampling studies by geologic formation. Air sampling has been performed in various UOG sites in the US. The most commonly sampled site in studies identified by our search was the Barnett Shale located in TX. The least frequently studied were Eagle Ford Shale, Fayetteville Shale, Haynesville Shale, and Powder River Basin. TX, Texas; AR, Arkansas; OK, Oklahoma; LA, Louisiana; MT, Montana; WY, Wyoming; NY, New York; MD, Maryland; PA, Pennsylvania; WV, West Virginia; OH, Ohio; VA, Virginia; KY, Kentucky; TN, Tennessee; CO, Colorado; ND, North Dakota; UT, Utah; KS, Kansas; NE, Nebraska; UOG, unconventional oil and gas

Table 1 lists the 48 citations of the articles that measured air pollutants on or near sites of UOG production. A distribution of the studies measuring UOG air pollutants in sites across the US is shown in Fig. 1. The majority of studies were done on the Barnett Shale in Texas (11 studies). The least studied were Eagle Ford Shale in Texas, Haynesville Shale in Louisiana, Arkansas, and Texas, Fayetteville Shale in Arkansas, and Powder River Basin in Montana and Wyoming, all with only one study each. One hundred six chemicals were detected in two or more of the 48 studies that measured air pollutants near UOG sites and another 115 were detected only once (see Additional file 1: Table S4 for full list of detected chemicals). These chemicals represented a variety of classes including alkanes, alkenes, alkynes, aromatics, aldehydes and polycyclic aromatic hydrocarbons (PAHs). Twenty chemicals were detected in 10 or more studies with ethane and benzene being the most detected, appearing in 56% and 54% of studies, respectively. Fifty-four chemicals were detected in 3-9 studies and 147 were detected in 2 or fewer.

**Table 1:** List of citations for UOG air papers

Author	Title	Sampling Location (Geologic Formation)
Brantley, HL. et al., 2015 [70]	Assessment of volatile organic compound and hazardous air pollutant emissions from oil and natural gas well pads using mobile remote and on-site direct measurements	Denver-Julesburg
Colborn, T. et al., 2014 [1]	An exploratory study of air quality near natural gas operations	Piceance
Eapi, GR. et al., 2014 [71]	Mobile measurement of methane and hydrogen sulfide at natural gas production site fence lines in the Texas Barnett Shale	Barnett
Eisele, AP. et al., 2016 [72]	Volatile organic compounds at two oil and natural gas production well pads in Colorado and Texas using passive samplers	Barnett; Denver-Julesburg
Esswein, EJ. et al., 2014 [73]	Evaluation of some potential chemical exposure risks during flowback operations in unconventional oil and gas extraction: Preliminary results	Denver-Julesburg; Green River; Piceance
Field, RA. et al., 2015 [20]	Influence of oil and gas field operations on spatial and temporal distributions of atmospheric non-methane hydrocarbons and their effect on ozone formation in winter	Green River
Field, RA. et al., 2015 [74]	Distributions of air pollutants associated with oil and natural gas development measured in the Upper Green River Basin of Wyoming	Green River
Gilman, JB. et al., 2013 [2]	Source signature of volatile organic compounds from oil and natural gas operations in northeastern Colorado	Denver-Julesburg
Goetz, JD. et al., 2015 [75]	Atmospheric emission characterization of Marcellus Shale natural gas development sites	Marcellus
Helmig, D. et al., 2014 [3]	Highly elevated atmospheric levels of volatile organic compounds in the Uintah Basin, Utah	Uintah
Katzenstein, AS. et al., 2003 [76]	Extensive regional atmospheric hydrocarbon pollution in the southwestern United States	Not reported
Koss, AR. et al., 2015 [77]	Photochemical aging of volatile organic compounds associated with oil and natural gas extraction in the Uintah Basin, UT, during a wintertime ozone formation event	Uintah
Lan, X. et al., 2015 [78]	Atmospheric Mercury in the Barnett Shale Area, Texas: Implications for emissions from oil and gas processing	Barnett
Lee, L. et al., 2015 [79]	Particulate organic nitrates observed in an oil and natural gas production region during wintertime	Uintah
Li, C. et al., 2016 [80]	Satellite observation of pollutant emissions from gas flaring activities near the Arctic	Bakken
Li, R. et al., 2014 [81]	Measurements of hydrogen sulfide (H2S) using PTR-MS: Calibration, humidity dependence, inter-comparison and results from field studies in an oil and gas production region	Uintah
Lyman, S. and Tran, T., 2015 [82]	Inversion structure and winter ozone distribution in the Uintah Basin, Utah, USA	Uintah
Macey, GP. et al., 2014 [4]	Air concentrations of volatile compounds near oil and gas production: a community-based exploratory study	Denver-Julesburg; Fayetteville; Green River; Marcellus; Powder River; Utica
McKenzie, LM. et al., 2012 [31]	Human health risk assessment of air emissions from development of unconventional natural gas resources	Piceance
Olaguer, EP. et al., 2015 [83]	Updated methods for assessing the impacts of nearby gas drilling and production on neighborhood air quality and human health	Eagle Ford
Oltmans, S. et al., 2014 [84]	Anatomy of wintertime ozone associated with oil and natural gas extraction activity in Wyoming and Utah	Green River; Uintah
Omara, M. et al., 2016 [85]	Methane emissions from conventional and unconventional natural gas production sites in the Marcellus Shale basin	Marcellus
Paulik, LB. et al., 2016 [59]	Emissions of polycyclic aromatic hydrocarbons from natural gas extraction into air	Utica
Peischl, J. et al., 2015 [86]	Quantifying atmospheric methane emissions from oil and natural gas production in the Bakken Shale region of North Dakota	Fayetteville; Haynesville; Marcellus
Pekney, NJ. et al., 2014 [87]	Measurement of atmospheric pollutants associated with oil and natural gas exploration and production activity in Pennsylvania's Allegheny National Forest	Marcellus
Petron, G. et al., 2012 [88]	Hydrocarbon emissions characterization in the Colorado Front Range: A pilot study	Denver-Julesburg
Petron, G. et al., 2014 [89]	A new look at methane and nonmethane hydrocarbon emissions from oil and natural gas operations in the Colorado Denver-Julesburg Basin	Denver-Julesburg
Prenni, AJ. et al., 2016 [90]	Oil and gas impacts on air quality in federal lands in the Bakken region: An overview of the Bakken Air Quality Study and first results	Bakken
Rappengluck, B. et al., 2014 [5]	Strong wintertime ozone events in the Upper Green River basin, Wyoming	Green River
Rich, A. et al., 2014 [16]	An exploratory study of air emissions associated with shale gas development and production in the Barnett Shale	Barnett
Rich, AL. and Orimoloye, HT., 2016 [91]	Elevated atmospheric levels of benzene and benzene-related compounds from unconventional shale extraction and processing: Human health concern for residential communities	Barnett
Roscioli, JR. et al., 2015 [15]	Measurements of methane emissions from natural gas gathering facilities and processing plants: Measurement methods	Not reported
Rutter, AP. et al., 2015 [92]	Sources of air pollution in a region of oil and gas exploration downwind of a large city	Barnett
Schnell, RC. et al., 2009 [93]	Rapid photochemical production of ozone at high concentrations in a rural site during winter	Green River
Schwarz, JP. et al., 2015 [94]	Black carbon emissions from the Bakken oil and gas development region	Bakken
Smith, ML. et al., 2015 [95]	Airborne ethane observations in the Barnett Shale: Quantification of ethane flux and attribution of methane emissions	Barnett
Swarthout, RF. et al., 2013 [96]	Volatile organic compound distributions during the NACHTT campaign at the Boulder Atmospheric Observatory: Influence of urban and natural gas sources	Denver-Julesburg
Swarthout, RF. et al., 2015 [97]	Impact of Marcellus Shale natural gas development in southwest Pennsylvania on volatile organic compound emissions and regional air quality	Marcellus
Thompson, CR. et al., 2014 [98]	Influence of oil and gas emissions on ambient atmospheric non-methane hydrocarbons in residential areas of Northeastern Colorado	Denver-Julesburg
Townsend-Small, A. et al., 2015 [99]	Integrating source apportionment tracers into a bottom-up inventory of methane emissions in the Barnett Shale hydraulic fracturing region	Barnett
Vinciguerra, T. et al., 2015 [100]	Regional air quality impacts of hydraulic fracturing and shale natural gas activity: Evidence from ambient VOC observations	Marcellus
Warneke, C. et al., 2014 [6]	Volatile organic compound emissions from the oil and natural gas industry in the Uintah Basin, Utah: Oil and gas well pad emissions compared to ambient air composition	Uintah
Warneke, C. et al., 2015 [101]	PTR-QMS versus PTR-TOF comparison in a region with oil and natural gas extraction industry in the Uintah Basin in 2013	Uintah
Weyant, CL. et al., 2016 [102]	Black carbon emissions from associated natural gas flaring	Bakken
Yacovitch, TI. et al., 2015 [103]	Mobile laboratory observations of methane emissions in the Barnett Shale region	Barnett
Yuan, B. et al., 2015 [104]	Airborne flux measurements of methane and volatile organic compounds over the Haynesville and Marcellus Shale gas production regions	Haynesville; Marcellus
Zavala-Araiza, D. et al., 2014 [105]	Atmospheric hydrocarbon emissions and concentrations in the Barnett Shale natural gas production region	Barnett
Zielinska, B. et al., 2014 [7]	Impact of emissions from natural gas production facilities on ambient air quality in the Barnett Shale area: A pilot study	Barnett

**Fig. 2:**
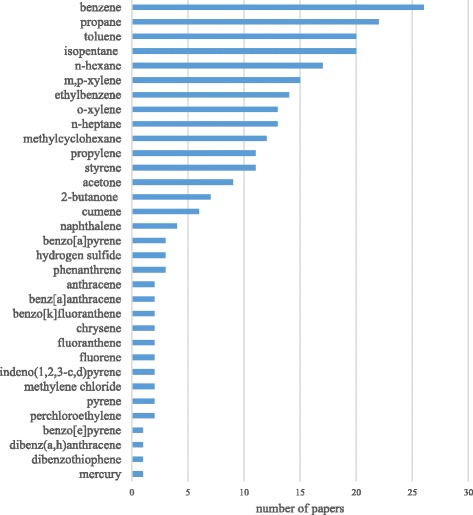
Potentially endocrine active chemicals and the number of studies that identified them near UOG sites. The figure shows the 34 chemicals (with m-xylene and p-xylene counted separately) that were identified as having evidence of endocrine active properties and the number of times they were detected in the air sampling papers included in this study. The graph show that the BTEX compounds (benzene, toluene, ethylbenzene, xylenes) were among the most frequently detected, and the polycyclic aromatic hydrocarbons (PAHs) were less frequently detected in air samples

The list of chemicals detected near UOG activity was cross-referenced with the TEDX List of Potential Endocrine Disruptors. Twenty-six were already identified and listed in the TEDX List of Potential Endocrine Disruptors [49]. There were 15 additional chemicals that were reported as being detected in 10 or more UOG studies, but that were not currently included in the TEDX List of Potential Endocrine Disruptors that were searched. A chemical’s absence on the TEDX List of Potential Endocrine Disruptors does not necessarily mean there is no evidence for endocrine activity. Rather, it is possible that the literature available for that chemical has not yet been investigated for endocrine activity. The searches of PubMed for the 15 frequently detected chemicals yielded eight with evidence from the literature indicating at least one study had shown the chemicals to be endocrine active (including findings related to potential endocrine activity). Those chemicals were m-xylene, p-xylene, o-xylene, methylcyclohexane, n-heptane, isopentane, propane, propylene. There were no studies that evaluated the endocrine activity of ethane, n-butane, isobutane, ethylene, cyclohexane and acetylene found in our searches. In studies identified by our search that assessed the effects of n-pentane [53] and n-octane [54] endocrine activity was not shown. Table 2 characterizes possible endocrine activities for the individual chemicals. The studies listed in this table tested more direct indicators of endocrine activity such as estrogenic, androgenic, thyroidogenic, progestrogenic, glucocorticodogenic, and steroidogenic activities. In Table 3, chemicals identified as having evidence of physiological activity that may be linked to endocrine disruption are shown. This includes evaluations of reproduction, aryl hydrocarbon signaling, development, neurophysiology, and other endocrine related effects. Notably, a few of these air pollutants (e.g., benzene, n-hexane, and isopentane) may impact less commonly evaluated endocrine related endpoints such as insulin signaling and adrenal physiology (see Table 3). Roughly half of the chemicals in Tables 2 and 3 are PAHs, although it should be noted that few studies detected PAHs near UOG (see Fig. 2). Single ring aromatics such as benzene, toluene, ethylbenzene, xylene, and styrene are also shown in Tables 2 and 3 with evidence suggesting possible estrogenic, androgenic, reproductive, and developmental effects. Styrene seems to be of particular concern because in addition to the aforementioned evidence of endocrine activity it also appears to have evidence for glucocorticodogenic, thyroidogenic, and progestrogenic, activity and alterations of steroidgenesis.

**Table 2:** Selected studies indicating endocrine activity*

Chemical	Estrogenic	Androgenic	Thyroidogenic	Progestrogenic	Glucocorticodogenic	Steroidogenesis
benzene^^^	Kassotis et al., 2015 [41]	Kassotis et al., 2015 [41]				
toluene^	Kassotis et al., 2015 [41]	Kassotis et al., 2015 [41]				
n-hexane^	Kassotis et al., 2015 [41]	Kassotis et al., 2015 [41]				
p-xylene^						Ungvary et al., 1981 [106]
ethylbenzene^	Kassotis et al., 2015 [41]	Kassotis et al., 2015 [41]				
xylenes^	Kassotis et al., 2015 [41]	Kassotis et al., 2015 [41]		Kassotis et al., 2015 [41]		
methylcyclohexane						Kim et al., 2011 [107]
styrene^	Kassotis et al., 2014 [10]; Kassotis et al., 2015 [41]	Kassotis et al., 2014 [10]; Kassotis et al., 2015 [41]	Kassotis et al., 2015 [41]	Kassotis et al., 2015 [41]	Kassotis et al., 2015 [41]	Takao et al., 2000 [108]
cumene^	Kassotis et al., 2014 [10]; Kassotis et al., 2015 [41]	Kassotis et al., 2014 [10]; Kassotis et al., 2015 [41]		Kassotis et al., 2015 [41]		
benzo[a]pyrene^	Vondracek et al., 2002 [109]	Vinggaard et al., 2000 [110]				Monteiro et al., 2000a [111]
naphthalene^	Kassotis et al., 2014 [10]; Kassotis et al., 2015 [41]	Kassotis et al., 2014 [10]; Kassotis et al., 2015 [41]	Kassotis et al., 2015 [41]	Kassotis et al., 2015 [41]	Kassotis et al., 2015 [41]	Evanson and Van Der Kraak, 2001 [112]; Pollino et al., 2009 [113]
phenanthrene^^^	Vondracek et al., 2002 [109]					Monteiro et al., 2000a [111]; Monteiro et al., 2000b [114]
anthracene^^^	Vondracek et al., 2002 [109]					
benz[a]anthracene^^^	Vondracek et al., 2002 [109]	Vinggaard et al., 2000 [110]				
chrysene^^^		Vinggaard et al., 2000 [110]				Monteiro et al., 2000a [111]; Monteiro et al., 2000b [114]
fluoranthene^^^	Vondracek et al., 2002 [109]	Vinggaard et al., 2000 [110]; Araki et al., 2005 [115]				
fluorene^^^	Vondracek et al., 2002 [109]					
pyrene^^^	Vondracek et al., 2002 [109]					
dibenz(a,h)anthracene^^^		Vinggaard et al., 2000 [110]				
dibenzothiophene^^^	Brinkmann et al., 2014 [116]; Petersen and Tollefsen, 2011 [117]					
mercury^			Barregard et al., 1994 [118]			

Twenty-one air pollutants had evidence indicating that they impact hormone production, mimic hormones, or inhibit hormone signaling. There were 19 chemicals listed on the TEDX List of Potential Endocrine Disruptors and two that were identified via PubMed searches of frequently detected UOG air pollutants. The studies listed in the table tested estrogenic, androgenic, thyroidogenic, progestrogenic, glucocorticodogenic, and steroidogenic activity in various manners including: *in vitro* steroidogenesis, receptor mediated reporter gene activity, vitellogenin induction assays, and epidemiological, *in vivo* and *ex vivo* experimental animal assessments. ^hazardous air pollutant (HAP) * Note: all possible endocrine activities for the individual chemicals are not described.

**Table 3:** Selected studies demonstrating effects potentially related to endocrine disruption*

Chemical	Reproductive	Aryl hydrocarbon receptor signaling	Developmental	Neurophysiological	Other evidence of endocrine activity
benzene^^^	Xu et al., 1998 [119]		Brown-Woodman et al., 1994 [120]		^a^Choi et al., 2014 [121]
propane	McKee et al., 2014 [122]				
toluene^^^	Ono et al., 1996 [123]		Brown-Woodman et al., 1994 [120]		
isopentane					^b^Yu et al., 2011 [124]
n-hexane^^^	Nylén et al., 1989 [125]				^c^Zorad et al., 1987 [126]
p-xylene^^^			Ungvary and Tatrai, 1985 [127]		
m-xylene^^^			Ungvary and Tatrai, 1985 [121]		
ethylbenzene^^^			Ungvary and Tatrai, 1985 [127]		^d^National Toxicology Program. 1999 [128]
o-xylene^^^			Ungvary and Tatrai, 1985 [127]		
xylenes^^^			Brown-Woodman et al., 1994 [120]		
methylcyclohexane	Kim et al., 2011 [107]				
n-heptane					^c^Zorad et al., 1987 [126]
propylene	Quest et al., 1984 [129]				Quest et al., 1984 [129]
styrene^^^			Brown-Woodman et al., 1994 [120]	Zaidi et al., 1985 [130]; Mutti et al., 1984 [131]	
acetone				Mitran et al., 1997 [132]	
2-butanone^^^				Mitran et al., 1997 [132]	
benzo[a]pyrene^^^	Thomas, 1990 [133]	Machala et al., 2001 [134]; Vinggaard et al., 2000 [110]			
hydrogen sulfide	Xu et al., 1998 [119]				
naphthalene^^^	Sarojini et al., 1995 [135]				
phenanthrene^^^	Evans and Nipper, 2007 [136]				
anthracene^^^	Hall and Oris, 1991 [137]				
benz[a]anthracene^^^		Machala et al., 2001 [134]; Vinggaard et al., 2000 [110]			^e^Benisek et al., 2011 [138]
benzo[k]fluoranthene^^^		Machala et al., 2001 [134]			
chrysene^^^		Machala et al., 2001 [134]			
fluoranthene^^^		Machala et al., 2001 [134]			
fluorene^^^			Incardona et al., 2004 [139]		
indeno(1,2,3-c,d)pyrene^^^		Machala et al., 2001 [134]			
methylene chloride^^^				Moser et al., 1995[140]	
pyrene^^^		Machala et al., 2001 [134]			
benzo[e]pyrene^^^		Machala et al., 2001 [134]			
dibenz(a,h)anthracene^^^		Machala et al., 2001 [134]; Vinggaard et al., 2000 [110]			
dibenzothiophene^^^			Incardona et al., 2004 [139]		
perchloroethylene^^^	Carney et al., 2006 [141]		Fredriksson et al., 1993 [142]	Fredriksson et al., 1993 [142]; Honma et al., 1980 [143]; Shafer et al., 2005 [144]	^b^National Toxicology Program, 1986 [145]

Thirty-three air pollutants had evidence indicating they impacted processes and systems that are modulated by endocrine signaling. There were 25 chemicals listed on the TEDX list of Potential endocrine disruptors and eight that were identified via PubMed searches of frequently detected UOG air pollutants. The studies listed in the table tested aryl hydrocarbon signaling, reproductive, developmental, neurophysiological, and other endocrine related effects in epidemiological, *in vivo* and *ex vivo* experimental animal assessments, *in vitro* embryonic culture and receptor mediated reporter gene activity assays. ^a^ insulin resistance; ^b^ adrenal physiology; ^c^ insulin binding; ^d^ hyperplasia of pituitary and thyroid; ^e^ retinoic acid signaling; ^^^hazardous air pollutant (HAP). * Note that all potentially related endocrine impacts for the individual chemicals are not described

In Fig. 2, the air sampling data (Table 1) was combined with the data that assessed possible endocrine activity (Tables 2 and 3). The chemicals identified as potentially endocrine active are listed along with the number of studies that detected them in air near sites of UOG activity. This list included 34 chemicals with m-xylene and p-xylene counted separately, however they are combined (i.e., m,p-xylene) for the number of papers that detected them in the air to be consistent with how they are reported in that literature. In total, this list includes the 26 chemicals that were already on the TEDX List of Potential Endocrine Disruptors and the eight frequently detected UOG associated air pollutants that were found to have potential endocrine activity. Benzene, toluene, ethylbenzene, and xylenes (BTEX) were detected more frequently than PAHs and heavy metals such as mercury.

## Discussion

Our study revealed more than 200 air chemicals in association with UOG activity at sites in the US. We identified 26 as being on the TEDX list, which identifies chemicals with endocrine activity, and an additional eight of the most frequently detected air pollutants were identified as having potential endocrine activity. Endocrine activities included estrogenicity, androgenicity and altered steroidogenesis. In addition, we included evidence from studies assessing endpoints related to developmental, neurophysiological and reproductive changes commonly mediated by hormones [55].

The BTEX compounds were among the top 10 most detected chemicals across the studies in our sample. This is likely due to the existence of less expensive detection methods and their recognition as HAPs according to the United States Environmental Protection Agency (US EPA) [56]. The toxicity of the BTEX chemicals has been extensively studied with respect to respiratory, cardiovascular, neurological, and carcinogenic impacts, yet according to recent studies it is becoming apparent that they may also have impacts on endocrine function [41, 57]. Styrene, a structurally related compound, was also frequently detected and appears to have the ability to interfere with several endocrine pathways potentially resulting in alterations in development and neurophysiology. This compound has been studied extensively for cancer related outcomes and is “*reasonably anticipated to be a carcinogen*,” according to the National Toxicology Program [58]. Likewise, naphthalene is a possible carcinogen as well as a HAP [14] and appears to affect several different endocrine pathways. Few studies measured PAHs near UOG. One study that measured a wide array of PAHs in the air near UOG found increased concentrations at sites closest to active wells. These levels did not exceed EPA’s acceptable risk level for cancer, the only health effect addressed in the study [59]. In addition to carcinogenic properties, low level exposure to PAHs during prenatal development has been associated with delayed mental development, decreases in intelligence quotient (IQ), and childhood obesity [60–63]. Thus it is important to determine if they are pollutants commonly associated with UOG.

This study does not present a comprehensive review of research on the endocrine activity of compounds detected in the air near UOG. Rather, it serves to flag endocrine active compounds in order to inform future research on the potential health impacts of UOG. Further, some of the endocrine pathways have not been studied extensively and have not been replicated across models. In addition, some of the chemicals were not tested as inhalants in the studies we used to document endocrine disruption though this is the suspected primary route of exposure for the air pollutants evaluated. Our study only surveyed studies performed in the US, therefore it is possible that had we included studies from other countries the patterns of chemical detections may have differed. We also excluded foreign language studies, for lack of interpretive resources.

The review is limited by the fact that the primary studies routinely used standardized protocols (e.g., EPA Method TO-12, American Standard Test Method [ASTM] D-1357-95) that were likely informed by the US EPA’s HAPs list, which would lead to a bias in terms of which chemicals are tested for and thus detected. In other words, there may be more chemicals present near UOG, particularly proprietary chemicals used in drilling and hydraulic fracturing, that have not been assessed near well pads or other facilities. Therefore, the present review is also limited in identifying other potentially endocrine active chemicals that have not yet been quantified or have been detected less frequently.

The published literature suggests a relationship between proximity to and/or density of UOG development and adverse health impacts in humans and wildlife, including outcomes that are a result of exposure to endocrine active compounds [10, 35–38, 40, 64]. Our survey of the literature, while limited, supports these observations given that some of the air pollutants identified near sites of UOG activity are potentially endocrine active. Due to the types and hazards of the chemicals identified, there is a need to pursue additional long-term studies in humans and wildlife that investigate endocrine mediated health outcomes in order to understand whether or not exposure to endocrine active air pollutants results in disease. However, these studies are time-consuming, and a delay in action may be considered unethical since it is already known that 28 chemicals identified in our study are HAPs (i.e., “are known to cause cancer or other serious health impacts [56]”) and several others have been studied thoroughly and identified as harmful to humans [65–68]. It was recently estimated that 17.6 million people in the US live within a mile of a well [69]. Thus, these populations may be exposed to air pollutants that have been linked to health impacts. It may be prudent to implement precautions similar to other industries that reduce exposure to air pollutants known to be health hazards.

For chemicals with sufficient bodies of literature but undefined hazard classifications, strategic execution of systematic reviews should follow as needed. These reviews would provide for a comprehensive analysis of the bodies of literature in order to determine confidence in the findings and/or potentially identify research gaps that might be addressed by more primary research. In addition, comprehensive environmental sampling of a broader array of chemicals (i.e., beyond HAPs) using novel laboratory techniques is necessary to establish if other air pollutants of concern are being emitted that are not included in standard testing protocols. Lastly, periodic updates to reviews, such as the present study, that assimilate new data are useful in characterizing the changing research landscape and can be used to redirect primary research efforts and policy actions as needed.

## Conclusions

The results of this study provide a basis for directing future primary research about the endocrine disrupting properties of air pollutants near UOG sites including exposure research in wildlife and humans. In addition, thoughtfully designed systematic reviews of the health impacts of specific chemicals should be conducted. Environmental testing for emerging chemicals of concern is also recommended.

In closing, there is evidence that individual air pollutants associated with UOG activity are endocrine active. Endocrine disruptors can have actions at low exposure concentrations, and exposures can lead to aberrant trajectories resulting in suboptimal developmental, behavioral, reproductive, and metabolic conditions. Yet, the magnitude of exposures specific to UOG, and the possible long-term health impacts, are not well understood. Further, several of the chemicals we identified are already designated by the US EPA as suspected or known carcinogens, are known to cause adverse developmental or reproductive effects, and are known for other toxicities (e.g., hearing loss, and nerve damage). Given the potential for health impacts and the lack of safety recommendations for many of the chemicals we identified, there is an urgent need to address these releases near human and wildlife populations.

## Additional file

Additional file 1: **Table S1.** Search terms used to identify air pollutants associated with UOG production. **Table S2.** PubMed search logic for chemicals with 10 or more detections from air studies that were not found on the TEDX List of Potential Endocrine Disruptors. **Table S3.** SWIFT search logic used to identify primary articles potentially describing ED activity. **Table S4.** List of chemicals reported as detected in air from 48 papers measuring air pollutants attributed to UOG activity. (DOCX 50 kb)

## Abbreviations

AR: Arkansas
ASTM: American Standard Test Method
BTEX: benzene, toluene, ethylbenzene, and xylenes
CO: Colorado
HAP: hazardous air pollutant
IQ: intelligence quotient
KS: Kansas
KY: Kentucky
LA: Louisiana
MD: Maryland
MT: Montana
ND: North Dakota
NE: Nebraska
NY: New York
OH: Ohio
OK: Oklahoma
PA: Pennsylvania
PAH: Polycyclic aromatic hydrocarbon
SWIFT: Sciome Workbench for Interactive computer-Facilitated Text-mining
TEDX: The Endocrine Disruption Exchange
TN: Tennessee
TX: Texas
UOG: unconventional oil and gas
US EPA: United States Environmental Protection Agency
US: United States
UT: Utah
VA: Virginia
VOC: volatile organic compounds
WV: West Virginia
WY: Wyoming
