# Supplementary material for: Exploring the endocrine activity of air pollutants associated with unconventional oil and gas extraction
Source: Environ Health. 2018 Mar 21;17:26. doi: 10.1186/s12940-018-0368-z (PMC5861625; doi:10.1186/s12940-018-0368-z)
Supplement: Supplementary file 1 — Table S1. Search terms used to identify air pollutants associated with UOG production. Table S2. PubMed search logic for chemicals with 10 or more detections from air studies that were not found on the TEDX List of Potential Endocrine Disruptors. Table S3. SWIFT search logic used to identify primary articles potentially describing ED activity. Table S4. List of chemicals reported as detected in air from 48 papers measuring air pollutants attributed to UOG activity. (DOCX 50 kb) [file 12940_2018_368_MOESM1_ESM.docx]

**Exploring the endocrine activity of air pollutants associated with unconventional oil and gas extraction.**

**Authors:**

**Ashley L. Bolden^1^**

**Kim Schultz^1^**

**Katherine E. Pelch^1^**

**Carol F. Kwiatkowski^1,2^**

**^1^The Endocrine Disruption Exchange (TEDX), www.TEDX.org, Eckert, Colorado, United States of America**

**^2^Department of Integrative Physiology, University of Colorado, Boulder, Colorado, United States of America**

**Corresponding Author**

Ashley L. Bolden (ashleybolden@tedx.org)

**Table S1. Search terms used to identify air pollutants associated with UOG production**

| **Terms related to hydraulic fracturing** | **Terms related to areas in the US with hydraulic fracturing activity** | **Terms related to air** |
| --- | --- | --- |
| fracking, hydraulic fracturing, unconventional | Niobrara, Mancos, Monterey, Barnett, Haynesville, Bakken, Marcellus, Utica, Fayetteville, Eagle Ford, Green River, Uintah, Piceance, San Juan, Denver-Julesburg, Powder River, Antrim | air, air pollution, emission, vent |

Search terms were identified across three categories: terms related to hydraulic fracturing, locations in the US with hydraulic fracturing, and air. These terms were then combined and used to complete electronic searches in PubMed and Web of Science intended to identify peer-reviewed articles that measured chemicals in the air near sites of unconventional oil and gas (UOG) activity.

**Table S2. PubMed search logic for chemicals with 10 or more detections from air studies that were not found on the TEDX List of Potential Endocrine Disruptors**

| **Chemical Name** | **Search Logic** |
| --- | --- |
| **ethane** | ethane OR bimethyl OR dimethyl OR methylmethane OR ethyl hydride OR 74-84-0[EC/RN Number] |
| **n-pentane** | pentane OR n-pentane OR 109-66-0[EC/RN Number] OR amyl hydride |
| **propane** | propane OR n-propane OR dimethylmethane OR propyl hydride OR 74-98-6[EC/RN Number] OR propyldihydride |
| **n-butane** | butane OR n-butane OR diethyl OR 106-97-8[EC/RN Number] OR methylethylmethane OR butyl hydride |
| **isopentane (2-methylbutane)** | 2-methylbutane OR isopentane OR isoamylhydride OR 2-methyl-butane OR  78-78-4[EC/RN Number] OR iso-pentane OR dimethylethylmethane OR ethyldimethylmethane OR 1,1,2-trimethylethane OR 1,1-dimethylpropane OR dimethyl-propane |
| **isobutane (2-methylpropane)** | isobutane OR 2-methylpropane OR 2-methyl-propane OR 75-28-5[EC/RN Number] OR trimethylmethane OR 1,1-dimethylethane OR dimethylethane OR methylpropane |
| **m,p xylene** | m-xylene OR 1,3-dimethylbenzene OR m-xylol OR 1,3-xylene OR m-dimethylbenzene OR meta-xylene OR 108-38-3[EC/RN Number] OR m-methyltoluene OR 3-xylene 1,3-dimethyl-benzene OR 1,3-dimethylbenzol OR 2,4-xylene OR m-dimethyl-benzene OR p-xylene OR 1,4-dimethylbenzene OR para-xylene OR 1,4-xylene OR p-dimethylbenzene OR p-methyltoluene OR p-xylol OR 106-42-3 OR 4-xylene OR 1,4-dimethyl-benzene |
| **o xylene** | o-xylene OR 1,2-dimethylbenzene OR o-xylol OR 1,2-xylene OR o-methyltoluene OR o-dimethylbenzene OR 3,4-xylene OR 2-xylene OR 95-47-6[EC/RN Number] OR 1,2-dimethyl-benzene, |
| **ethylene (ethene)** | ethylene OR ethene OR  acetene OR 74-85-1[EC/RN Number] |
| **methylcyclohexane** | methylcyclohexane OR hexahydrotoluene OR methyl-cyclohexane OR 108-87-2[EC/RN Number] OR cyclohexylmethane OR toluene hexahydride OR hexahydroxytoluene OR methyl cyclohexane OR methyl-cyclohexane |
| **n-heptane** | heptane OR n-heptane OR 142-82-5[EC/RN Number] OR dipropylmethane OR heptyl hydride OR dipropyl methane |
| **acetylene (ethyne)** | ethyne OR acetylene OR vinylene OR polyacetylene OR 74-86-2[EC/RN Number] |
| **n-octane** | octane OR n-octane OR 111-65-9[EC/RN Number] |
| **propylene (propene)** | propylene OR propene OR 1-propene OR methylethylene OR methylethene OR prop-1-ene 1-propylene OR 115-07-1[EC/RN Number] OR  n-propylene |
| **cyclohexane** | cyclohexane OR 110-82-7[EC/RN Number] OR hexamethylene OR hexanaphthene OR hexahydrobenzene |

Electronic searches were performed in PubMed for a subset of chemicals not listed on the TEDX list of potential endocrine disruptors as of October 2016. The above search logic was developed by identifying synonyms and other common names for the chemical including the CAS (Chemical Abstracts Service) registry number (indicated by EC/RN Number).

**Table S3. SWIFT search logic used to identify primary articles potentially describing ED activity**

| **General Endocrine** | tiab:"endocrin*" OR tiab:"hormon*" OR tiab:"*estrogen*" OR tiab:"*androgen*" OR tiab:"*testosterone*" OR tiab:"*thyr*" OR tiab:"adrenal" OR tiab:"*steroid*" OR tiab:"*cortico*" OR tiab:"cortisol" OR tiab:"pituitary" OR tiab:"thypothalam*" OR tiab:"hippocamp*" OR tiab:"pancrea*" OR tiab:"islet*" OR tiab:"insulin" OR tiab:"test?s" OR tiab:"testic*" OR tiab:"thymus" OR tiab:"thymic" OR tiab:"prostat*" OR tiab:"ovar*" OR tiab:"sertoli" OR tiab:"leydig" OR tiab:"follic*" OR tiab:"LH" OR tiab:"FSH" OR tiab:"lutenizing" OR tiab:"*natal*" OR tiab:"gestat*" OR tiab:"lacat*" OR tiab:"sperm*" OR tiab:"Oocyte*" OR tiab:"oogene*" OR tiab:"retinoi*" OR tiab:"reprod*" OR tiab:"fertil*" OR tiab:"ahr" OR tiab:"aryl hydrocarbon" OR tiab:"cyp" OR tiab:"cytochrome*" OR tiab:"PPAR*" OR tiab:"peroxisome proliferat*" |
| --- | --- |
| **General Endocrine Disruption** | mesh_mh:"endocrine disruptors" OR tiab: xenoestrogen* OR tiab:(endocrine AND disrupt*) OR tiab:(hormon* AND disrupt*) OR tiab:(EDCs) |
| **Estrogenic** | (mesh_mh:"estrogens" NOT mesh_mh: "estrogens esterified (USP)") OR tiab:"*estrogen*" OR mesh_mh:"aromatase" OR tiab:"cyp19" OR tiab:"cyp 19" OR tiab:"P450AROM" OR tiab:"cytochrome P450 19" OR tiab:"cytochrome P 450(AROM)" OR tiab:"Cytochrome P 450 CYP19" OR tiab:"Cytochrome P450 CYP19" OR tiab:"*estrogen synthase" OR tiab:"*estrogen synthetase" OR tiab:"androstenedione aromatase" OR suppchem: "GPER protein, human" OR suppchem: "GPR30 protein, rat" OR suppchem: "GPER protein, zebrafish" OR suppchem "GPER1 protein, xenopus" OR tiab: "G protein coupled *estrogen receptor*" OR tiab: "GPER" OR tiab: "GPR30" OR tiab: "GPR41" OR (tiab: "mER" AND tiab: "*estrogen*") OR suppchem:"estrogen receptor-related receptor beta" OR tiab:"*estrogen receptor-related receptor* beta" OR tiab:"ERR beta" OR tiab:"ERRbeta" OR tiab:"ERalpha" OR tiab:"ER alpha" OR tiab:"ER beta" OR tiab:"ERbeta" OR tiab:"*estrogen receptor*" OR mesh_mh:"receptors, estrogen" |
| **Androgenic** | mesh_mh:"androgens" OR tiab:"androgen*" OR tiab:"antiandrogen*" OR mesh_mh:"receptors, androgen" OR tiab:"5 alpha dihydrotestosterone receptor*" OR tiab:"androgen receptor*" OR tiab:"testosterone receptor*" OR (tiab:"AR*" AND tiab: "androgen") OR suppchem:"AR protein, human" OR tiab:"AR protein" OR mesh_mh:"3-oxo-5-alpha-steroid 4-dehydrogenase" OR tiab:"3 oxo 5 alpha Steroid 4 dehydrogenase" OR tiab:"testosterone 5 alpha reductase" OR tiab:"5 alpha reductase" OR tiab:"mAR" OR (tiab:"G" AND tiab:"protein-coupled" AND tiab:"androgen" AND tiab:"receptor") OR tiab:"5?DHT" |
| **Thyroidogenic** | mesh_mh:"thyroid gland" OR tiab:"thyroid gland" OR mesh_mh:"Thyroid diseases" OR mesh_mh:"Adenocarcinoma, Follicular" OR tiab:"goiter" OR tiab:"graves-disease*" OR tiab:"hyperthyroid*" OR tiab:"hypothyroid*" OR tiab:"thyroiditis" OR tiab:"thyrotoxicosis" OR tiab:"myxedema" OR tiab:"euthyroid" OR (mesh_mh:"Thyroid hormones" NOT mesh_mh:"thyroid (USP)") OR tiab:"dextrothyroxine" OR tiab:"diiodothyronine*" OR tiab:"diiodotryosine" OR tiab:"monoiodotyrosine" OR tiab:"thyronine*" OR tiab:"thyroxine" OR tiab:"triiodothyronine" OR mesh_mh:"Thyroid hormone resistance Syndrome" OR tiab:"Refetoff Syndrome" OR mesh_mh:"Receptors, thyroid hormone" OR tiab:"T3-receptor*" OR tiab:"T4-receptor*" OR tiab:"TR alpha" OR tiab:"Tralpha" OR tiab:"c-erbA" OR tiab:"THRA" OR tiab:"NR1A1*" OR tiab:"TR beta" OR tiab:"Trbeta" OR tiab:"thrb" OR tiab:"NR1A2" OR suppchem:"Sodium-iodide Symporter" OR tiab:"iodide transporter" OR tiab:"sodium iodide symporter" OR tiab:"sodium-iodide cotransporter" OR (tiab:"NIS" AND (tiab:"Protein" OR "symporter")) OR tiab:"SLC5A5" OR mesh_mh:"iodide peroxidase" OR tiab:"iodide peroxidase" OR tiab:"5' Deiodinase" OR tiab:"Thyroid peroxidase" OR tiab:"Iodotyrosine Deiodinase" OR tiab:"iodinase" OR tiab:"Iodotyrosine Deiodase" OR tiab:"Monodeiodinase" OR tiab:"Deiodinase" OR tiab:"Iodothyronine Deiodinase" OR suppchem:"Thyroid hormone-binding proteins" OR mesh_mh:"Thyroxine-Binding Proteins" OR mesh_mh:"Thyroglobulin" OR suppchem:"thyroglobulin receptor" OR tiab:"Thyroid hormone binding proteins" OR tiab:"Thyroxine Binding Proteins" OR tiab:"Thyroglobulin" OR tiab:"thyroid stimulating hormone" OR tiab:"TSH" OR mesh_mh:"receptors, thyrotropin" OR tiab:"thyrotropin" |

The above logic was designed for use in Sciome Workbench for Interactive Computer-Facilitated Text-mining (SWIFT)-Review using Medical Subject Headings (MeSH) terms and keywords to search the titles and abstracts (i.e., tiab) of records in order to identify those that evaluated endocrine or endocrine related endpoints following exposure to a chemical. Records from the chemical searches in PubMed were filtered with the above logic using SWIFT-Review.

**Table S4. List of chemicals reported as detected in air from 48 papers measuring air pollutants attributed to UOG activity**

| **Chemical Name** | **CAS** | **Times Detected** |
| --- | --- | --- |
| ethane | 74-84-0 | 27 |
| benzene | 71-43-2 | 26 |
| n-butane | 106-97-8 | 22 |
| n-pentane | 109-66-0 | 22 |
| propane | 74-98-6 | 22 |
| isopentane (2-methylbutane) | 78-78-4 | 20 |
| toluene | 108-88-3 | 20 |
| isobutane (2-methylpropane) | 75-28-5 | 18 |
| n-hexane | 110-54-3 | 17 |
| m,p-xylene | 108-38-3/106-42-3 | 15 |
| ethylbenzene | 100-41-4 | 14 |
| ethylene (ethane) | 74-85-1 | 13 |
| n-heptane | 142-82-5 | 13 |
| o-xylene | 95-47-6 | 13 |
| acetylene (ethyne) | 74-86-2 | 12 |
| methylcyclohexane | 108-87-2 | 12 |
| n-octane | 111-65-9 | 11 |
| propylene (propene) | 115-07-1 | 11 |
| styrene | 100-42-5 | 11 |
| cyclohexane | 110-82-7 | 10 |
| acetone | 67-64-1 | 9 |
| isohexane (2-methylpentane) | 107-83-5 | 9 |
| isoprene | 78-79-5 | 9 |
| methylcyclopentane | 96-37-7 | 9 |
| 1,2,4-trimethylbenzene | 95-63-6 | 8 |
| carbon monoxide | 630-08-0 | 8 |
| n-nonane | 111-84-2 | 8 |
| 1,3,5-trimethylbenzene | 108-67-8 | 7 |
| 2-butanone | 78-93-3 | 7 |
| 3-methylpentane | 96-14-0 | 6 |
| acetaldeyde | 75-07-0 | 6 |
| cumene (isopropylbenzene) | 98-82-8 | 6 |
| cyclopentane | 287-92-3 | 6 |
| isooctane (2,2,4-trimethylpentane) | 540-84-1 | 6 |
| methanol | 67-56-1 | 6 |
| n-propylbenzene | 103-65-1 | 6 |
| 1,2,3-trimethylbenzene | 526-73-8 | 5 |
| 1-butene | 106-98-9 | 5 |
| 2,2-dimethylbutane | 75-83-2 | 5 |
| 2-methylheptane | 592-27-8 | 5 |
| 2-methylhexane | 591-76-4 | 5 |
| 3-methylheptane | 589-81-1 | 5 |
| a-pinene | 80-56-8 | 5 |
| carbon dioxide | 124-38-9 | 5 |
| formaldehyde | 50-00-0 | 5 |
| trans-2-butene | 68956-54-7 | 5 |
| 1,3-butadiene | 106-99-0 | 4 |
| 1-pentene | 109-67-1 | 4 |
| 2,4-dimethylpentane | 108-08-7 | 4 |
| 3-methylhexane | 589-34-4 | 4 |
| cis-2-butene | 590-18-1 | 4 |
| ethanol | 64-17-5 | 4 |
| methacrolein | 78-85-3 | 4 |
| m-ethyltoluene | 620-14-4 | 4 |
| naphthalene | 91-20-3 | 4 |
| o-ethyltoluene | 611-14-3 | 4 |
| p-ethyltoluene | 622-96-8 | 4 |
| 1-hexene | 592-41-6 | 3 |
| 2,3-dimethylbutane | 79-29-8 | 3 |
| 2,3-dimethylpentane | 565-59-3 | 3 |
| 2-methyl-2-butene | 513-35-9 | 3 |
| benzo(g,h,i)perylene | 191-24-2 | 3 |
| benzo[a]pyrene | 50-32-8 | 3 |
| b-pinene | 127-91-3 | 3 |
| cis-2-pentene | 627-20-3 | 3 |
| d-limonene | 5989-27-5 | 3 |
| hydrogen sulfide | 7783-06-4 | 3 |
| n-decane | 124-18-5 | 3 |
| n-dodecane | 112-40-3 | 3 |
| n-undecane | 1120-21-4 | 3 |
| phenanthrene | 85-01-8 | 3 |
| propionaldehyde | 123-38-6 | 3 |
| propyne | 74-99-7 | 3 |
| trans-2-pentene | 68956-55-8 | 3 |
| 1,4-dichlorobenzene | 106-46-7 | 2 |
| 1-methyl propenylbenzene | 2082-61-3 | 2 |
| 2,3,4-trimethylpentane | 565-75-3 | 2 |
| 2-methyl propenylbenzene | 768-49-0 | 2 |
| 2-methyl-1-butene | 563-46-2 | 2 |
| anthracene | 120-12-7 | 2 |
| benz[a]anthracene | 56-55-3 | 2 |
| benzo(b)fluoranthene | 205-99-2 | 2 |
| benzo[k]fluoranthene | 207-08-9 | 2 |
| butyraldehyde | 123-72-8 | 2 |
| camphene | 79-92-5 | 2 |
| carbon disulfide | 75-15-0 | 2 |
| carbonyl sulfide (COS) | 463-58-1 | 2 |
| chrysene | 218-01-9 | 2 |
| cis-2-hexene | 7688-21-3 | 2 |
| dichlorodifluoromethane | 75-71-8 | 2 |
| fluoranthene | 206-44-0 | 2 |
| fluorene | 86-73-7 | 2 |
| fluorine | 86-73-7 | 2 |
| hexaldehyde | 66-25-1 | 2 |
| indeno(1,2,3-c,d)pyrene | 193-39-5 | 2 |
| m-diethylbenzene | 141-93-5 | 2 |
| methyl- methylethyl benzene/methylcumene | 19-87-6 | 2 |
| methylene chloride | 75-09-2 | 2 |
| neopentane (2,2 dimethylpropane) | 463-82-1 | 2 |
| p-diethylbenzene | 105-05-5 | 2 |
| perchloroethylene | 127-18-4 | 2 |
| pyrene | 129-00-0 | 2 |
| sulfur dioxide | 7446-09-5 | 2 |
| tetrahydrofuran | 109-99-9 | 2 |
| trans-2-hexene | 4050-45-7 | 2 |
| trichlorofluoromethane | 75-69-4 | 2 |
| 1‐ethyl‐3‐methylbenzene/1-ethyl-4-methylbenzene | 622-96-8/ 620-14-4 | 1 |
| 2-methyl-3-buten-2-ol (MBO) | 115-18-4 | 1 |
| 1,1,2,2,-tetrachloroethane | 79-34-5 | 1 |
| 1,2 diethylbenzene | 135-01-3 | 1 |
| 1,2,4-trichlorobenzene | 120-82-1 | 1 |
| 1,2-dichloroethane | 107-06-2 | 1 |
| 1,3-cyclohexadiene | 592-57-4 | 1 |
| 1,7-dimethylphenanthrene | 483-87-4 | 1 |
| 1-butanol | 71-36-3 | 1 |
| 1-ethyl-4-methylcyclohexane | 3728-56-1 | 1 |
| 1-methyl-1H indene | 767-59-9 | 1 |
| 1-methylnaphthalene | 90-12-0 | 1 |
| 1-methylphenanthrene | 832-69-9 | 1 |
| 1-methylpyrene | 2381-21-7 | 1 |
| 1-propynyl benzene | 673-32-5 | 1 |
| 1-R-alpha-pinene/2-Pinene/ 2.6.6Trimethylbichyclo[3.3.1]hept-2-ene | 7785-70-8 | 1 |
| 2,2,-dimethylhexane | 590-73-8 | 1 |
| 2-ethylthiophene | 872-55-9 | 1 |
| 2-methylanthracene | 613-12-7 | 1 |
| 2-methylnaphthalene | 91-57-6 | 1 |
| 2-methylphenanthrene | 2531-84-2 | 1 |
| 2-pentyl nitrate (2-PenONO2) | 21981-48-6 | 1 |
| 3,6-dimethylphenanthrene | 1576-67-6 | 1 |
| 3-carene | 13466-78-9 | 1 |
| 3-methylphenanthrene | 832-71-3 | 1 |
| 3-pentyl nitrate (3-PenONO2) | 82944-59-0 | 1 |
| 4-methyl-2-pentanone | 108-10-1 | 1 |
| 4-methylpyrene | 3353-12-6 | 1 |
| 7,12-dimethylbenz(a)anthracene | 57-97-6 | 1 |
| 9-methylanthracene | 779-02-2 | 1 |
| 9-methylphenanthrene | 883-20-5 | 1 |
| 9-phenylanthracene | 602-55-1 | 1 |
| acenaphthene | 83-32-9 | 1 |
| acenaphthylene | 208-96-8 | 1 |
| acetonitrile | 75-05-8 | 1 |
| benzaldehyde | 100-52-7 | 1 |
| benzo(b)chrysene | 214-17-5 | 1 |
| benzo(c)phenanthrene | 195-19-7 | 1 |
| benzo(g,h,i)fluoranthene | 203-12-3 | 1 |
| benzo(j)fluoranthene | 205-82-3 | 1 |
| benzo[c]fluorene | 205-12-9 | 1 |
| benzo[e]pyrene | 192-97-2 | 1 |
| benzonaphthothiophene | 61523-34-0 | 1 |
| chlorobenzene | 108-90-7 | 1 |
| chloroform | 67-66-3 | 1 |
| chloromethane | 74-87-3 | 1 |
| cis-1,3-dimethylcyclohexane | 638-04-0 | 1 |
| cis-3-hexene | 7642-09-3 | 1 |
| coronene | 191-07-1 | 1 |
| crotonaldehyde | 123-73-9 | 1 |
| cyclopenta(c,d)pyrene | 27208-37-3 | 1 |
| dibenz(a,h)anthracene | 53-70-3 | 1 |
| dibenzo(a,e)pyrene | 192-65-4 | 1 |
| dibenzo(a,i)pyrene | 189-55-9 | 1 |
| dibenzo(a,l)pyrene | 191-30-0 | 1 |
| dibenzo(ac)anthracene | 215-58-7 | 1 |
| dibenzo(ah)anthracene | 53-70-3 | 1 |
| dibenzothiophene | 132-65-0 | 1 |
| diemethyl pyridine | 108-48-5 | 1 |
| diethyl disulfide | 110-81-6 | 1 |
| diethyl sulfide | 352-93-2 | 1 |
| diethyl trisulfide | 3600-24-6 | 1 |
| difluorochloromethane | 75-45-6 | 1 |
| dimethyl disulfide | 624-92-0 | 1 |
| dimethyl sufide | 75-18-3 | 1 |
| dimethyl sulfate (DMS) | 77-78-1 | 1 |
| dimethyl trisulfide | 3658-80-8 | 1 |
| dinitrogen pentoxide (N2O5) | 10102-03-1 | 1 |
| dipropyl disulfide | 629-19-6 | 1 |
| ethyl acetate | 141-78-6 | 1 |
| ethyl mercaptan | 75-08-1 | 1 |
| ethyl methylethyl disulfide | 53966-36-2 | 1 |
| ethyl nitrate (EtONO2) | 109-95-5 | 1 |
| ethyl n-propyl disulfide | 30453-31-7 | 1 |
| ethylcyclohexane | 1678-91-7 | 1 |
| formic acid | 64-18-6 | 1 |
| glyoxal | 107-22-2 | 1 |
| g-terpinene | 99-85-4 | 1 |
| hexachlorobutadiene | 87-68-3 | 1 |
| indane | 496-11-7 | 1 |
| indeno[123-cd]fluoranthene | 193-43-1 | 1 |
| isobutyl mercaptan | 513-44-0 | 1 |
| isobutyl nitrate (2-BuONO2) | 542-56-3 | 1 |
| isopropanol | 67-63-0 | 1 |
| isopropyl mercaptan | 75-33-2 | 1 |
| isopropyl nitrate (2-PrONO2) | 1712-64-7 | 1 |
| mercury | 7439-97-6 | 1 |
| methyl cyclopentane | 96-37-7 | 1 |
| methyl ethyl disulphide | 20333-39-5 | 1 |
| methyl mercaptan | 74-93-1 | 1 |
| methyl n-butyl disulfide | 60779-24-0 | 1 |
| methyl nitrate (MeONO2) | 598-58-3 | 1 |
| methyl propyl disulfide | 2179-60-4 | 1 |
| methyl vinyl ketone | 78-94-4 | 1 |
| methylglyoxal | 78-98-8 | 1 |
| nitrate | 14797-55-8 | 1 |
| nitric acid | 7697-37-2 | 1 |
| nitrous acid | 7782-77-6 | 1 |
| nitryl chloride (ClNO2) | 13444-90-1 | 1 |
| n-propyl mercaptan | 107-03-9 | 1 |
| p-cymene | 99-87-6 | 1 |
| perylene | 198-55-0 | 1 |
| picene | 213-46-7 | 1 |
| propyl n-butyl disulfide | 1613-46-3 | 1 |
| propyl nitrate (1-PrONO2) | 627-13-4 | 1 |
| propynylbenzene | 673-32-5 | 1 |
| retene | 483-65-8 | 1 |
| tert-butyl mercaptan | 75-66-1 | 1 |
| tetrahydrothiophene | 110-01-0 | 1 |
| thieno[3,2] thiopene | 251-41-2 | 1 |
| thiophene | 110-02-1 | 1 |
| trans-1,2-dimethylcyclohexane | 6876-23-9 | 1 |
| trans-1,3- dimethylcyclohexane | 2207-03-6 | 1 |
| trichloroethene | 79-01-6 | 1 |
| triphenylene | 217-59-4 | 1 |
|  |  |  |

Initial list of individual chemicals detected in the 48 UOG air sampling studies. This list was cross-referenced with the TEDX List of Potential Endocrine Disruptors and was also used to identify the chemicals that were detected in greater than 10 studies. Exclusions included chemical concentrations that were measured in bulk (e.g., [NMHC], [VOC], and [C9-aromatics]), methane, ozone, NO_x_ (i.e., NO, NO2, etc.), particulate matter (i.e., PM2.5, PM10, PM0.5) and those that were unable to be CAS verified.
